# Supplementary material for: Epidemic spread simulation in an area with a high-density crowd using a SEIR-based model
Source: PLoS One. 2021 Jun 17;16(6):e0253220. doi: 10.1371/journal.pone.0253220 (PMC8211270; doi:10.1371/journal.pone.0253220)
Supplement: Attachment — Submitted filename: Response to Reviewer Comments_R1.pdf [file pone.0253220.s001.pdf]

Epidemic Spread Simulation in an Area with a High-density Crowd Using a SEIR-Based Model

(Manuscript ID: PONE-D-21-06411)

Response to Reviewer Comments,

We would like to thank the reviewers for their constructive comments, which have significantly improved the quality of the paper. We have revised the manuscript according to your constructive suggestions, where the corresponding changes to your comments have been highlighted in **RED** in the revised version of the manuscript. The corresponding responses to your insightful comments are attached as follows.

Reviewer #1

This manuscript adopts the traditional SEIR model to simulate epidemic spread in Tianyi station at Ningbo city. The authors have conducted lots of numerical simulation, The paper is well organized and it is a interesting topic. The proposed materials and methods are described in detail afterwards. ***In general, I think that the article fits well into the scope of target journal***. However, some revisions are required before the paper can be considered for publication.

1. Please give more explain for SEIR model, such as perturbation factor.

- **Thank you for your comments. The SEIR model was used to model passengers with four statuses: susceptible (S), exposed (E), infected (I), and recovered (R). Each variable represents the number of passengers in the corresponding group. The probability of being infected β represents the rate at which people move from S and I to R. Assuming that the total number of regions N remains unchanged and that N=S+E+I+R is satisfied, the differential equations are constructed as:**

$$\begin{cases} \frac{dS}{dt} = \mu(N-S) - \frac{\beta SI}{N} - \nu S \\ \frac{dE}{dt} = \frac{\beta SI}{N} - \mu E - \sigma E \\ \frac{dI}{dt} = \sigma E - \gamma I - \mu I \\ \frac{dR}{dt} = \gamma I - \mu R + \nu S \end{cases} \tag{1}$$

where v is the perturbation factor, μ is the natural mortality rate, β is the infection rate, γ is the recovery rate, and σ is the rate of moving from exposure to infection.

In addition, v is the perturbation factor, which indicates the ratio from the susceptible state S to the recovered state R. Its physical meaning is the

protective efficacy of an individual after vaccination, and a larger value indicates that government interventions are more effective, and vice versa.

2. The discussion in the body of the manuscript is abundant and lack of logic, which makes it harder to understand. The authors need to reorganize the structure significantly.

- **This is a good point. The discussion in the body of the manuscript has been modified, as follows,**
- **The factors influencing the spread of infectious diseases in urban rail transit stations mainly include the infectious disease transmission capacity and contact rate. With the increase of pedestrian contact rate and infectious disease transmission capacity, the more the maximum number of incubators and the maximum number of infectious disease illnesses, and the shorter the time to reach each peak.**
- **Hence, the controllable influences on the spread of infectious diseases can all be achieved by reducing the number of pedestrians in the station. Therefore, we propose a demand-responsive epidemic prevention strategy to control the number of people in stations according to the different severity of the epidemic, with specific measures including: subway reservation system, segregation in separate subway carriage, guidance and control of passenger flow, and adjustment of the departure interval of rail transportation, as shown in Fig 1.**

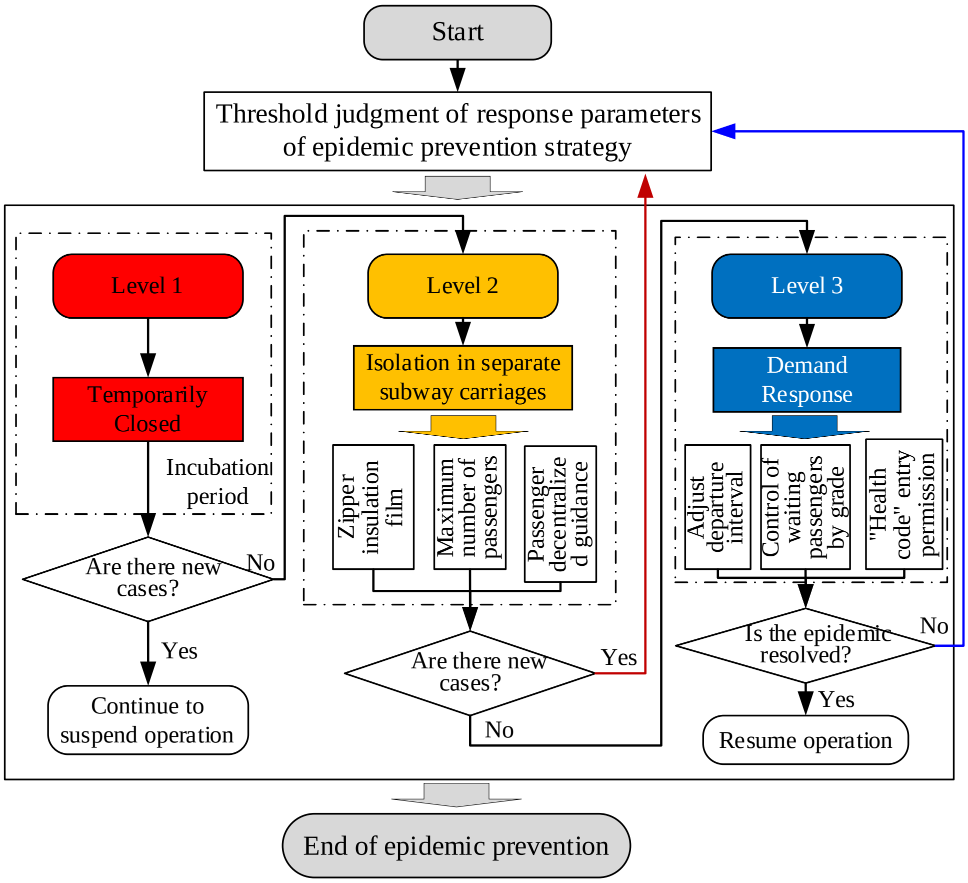

Figure 1 Three-Stage Epidemic Prevention Strategy of Urban Rail Transit

- **Level 1: Temporary stoppage of rail transit operation in the city. This strategy has the goal of cutting transmission pathways and is an intervention strategy for areas with the highest risk grade (red).**
- **Level 2: Isolation in separate cars in the second-highest-risk areas (orange). This strategy calls for the installation of zip-up isolation curtains between cars during the transitional epidemic period and has the goal of blocking the transmission of COVID-19 between cars. Specific measures will include: (1) the temporary installation of zip-up isolation curtains between different cars; (2) the adoption of a limit on the maximum number of persons in each car, such as by controlling seat occupancy at 30-50%; and (3) arranging for guide personnel to stand on both sides of each station platform and direct passengers to enter cars and take alternate seats in a dispersed manner. This strategy should be implemented in conjunction with a car numbering strategy.**
- **Level 3: Demand response strategy (blue). Dynamic adjustments will be made to operating plans in accordance with travel demand due to the three resumptions—resumption of workplace duties, resumption of work, and resumption of school—and big data from many sources in the public transportation system (including passenger ticket purchase data, station video monitoring data, platform passenger cell phone signal data, etc). There will be a few specific measures: (*a*) In the case of sections with a high load rate, shortening or adjustment of station stops and dynamic adjustment of junctions used by the trains and station-skipping operation plans, while balancing trains' load rates, will also meet passengers' travel demand. (*b*) To keep passengers from congregating in large numbers on station platforms, we propose that a multi-grade strategy be adopted to control waiting passengers. This multi-grade control strategy will be implemented in the safety inspection lanes, at the gates, and on the platform and will serve to disperse passenger flow, while ensuring that cars are not full. Workers at these important station control points will direct passengers in an orderly fashion to wait for and board trains in batches. (*c*) Implementation of a "health code" entry permission strategy at subway gates or station entry corridors. This health code will consist of green, red, and yellow codes and will facilitate management of passenger movement: Persons bearing a green code can scan the code to pass and will be permitted to take the subway. Persons displaying a red code must undergo 14 days of centralized or home isolation; after applying to have their health card punched for 14 consecutive days with normal health, they may receive a green code. Persons displaying a yellow code must undergo fewer than 7 days of centralized or home isolation, and after applying to have their health card punched for more than 7 consecutive days with normal health, they may receive a green code.**

3. Please give more explain for "1000 population" in your SEIR model.

- **Double thanks for your comment. The survey site located in Tianyi station at Ningbo Rail Transit Line 1, Ningbo, China, as shown in Figure 2. There are 4 entrances and exits at Tingyi Station, and 4 entrances and exits are open under normal circumstances. During the survey time, we took 15 minutes as a time interval to count the flow of pedestrians entering the station during working days and non-working days respectively, and finally summarized them in units of 1 hour, as shown in Table 1. Hence, for the convenience of simulation statistics, the number of simulated people per unit hour was set to 1000. Please find the detailed information on Line 6-12, Page 14.**

Table 1 Survey results in Tianyi station

**Weekend**		**Working-day**	
Time	Number	Time	Number
07:00—08:00	1031	07:00—08:00	1221
08:00—09:00	1131	08:00—09:00	1341
09:00—10:00	899	09:00—10:00	789
10:00—11:00	932	10:00—11:00	821
11:00—12:00	899	11:00—12:00	841
12:00—13:00	1021	12:00—13:00	1021
13:00—14:00	1031	13:00—14:00	942
14:00—15:00	947	14:00—15:00	854
15:00—16:00	943	15:00—16:00	765
16:00—17:00	1034	16:00—17:00	1147
17:00—18:00	1241	17:00—18:00	1353
18:00—19:00	931	18:00—19:00	865
19:00—20:00	945	19:00—20:00	798
20:00—21:00	849	20:00—21:00	776

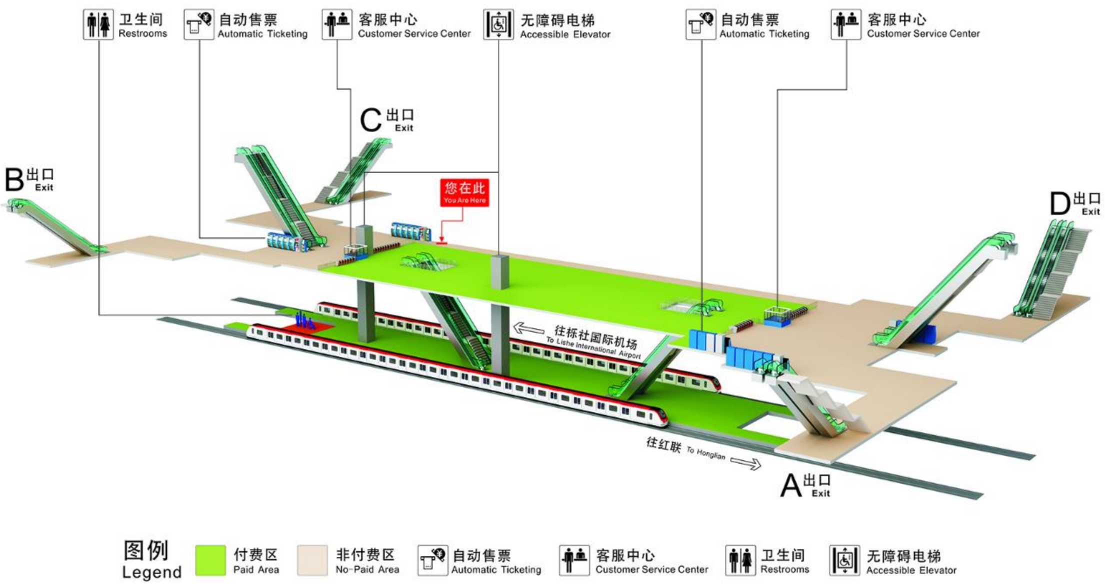

Figure 2 The survey site located in Tianyi station (Ningbo, China)

Reviewer #2

The paper simulated the spread of communicable diseases in an urban rail transit station. Data were collected by a field investigation in the city of Ningbo, China. A SEIR-based model was developed to simulate the spread of infectious diseases in Tianyi station, considering four groups of passengers (susceptible, exposed, infected, and recovered) and a 14-day incubation period. Based on the historical data of infectious diseases, the parameters of the SEIR infectious disease model were clarified, and a sensitivity analysis of the parameters was also performed. The paper is clearly written and easy to understand. My comments are pointed out as follows:

1. To enrich your bibliography, some important references about SEIR infectious disease model should be added.

   - **Thank you for your comments. As suggested by the reviewer, the related literatures have been added to the literature review chapter, as follows,**

[1] Stockmaier, S., Stroeymeyt, N., Shattuck, E. C., Hawley, D. M., Meyers, L. A., Bolnick, D. I. Infectious diseases and social distancing in nature. *Science*. 2021; *371*(6533): eabc8881.

[2] Heymann, D. L., Rodier, G. R. Hot spots in a wired world: WHO surveillance of emerging and re-emerging infectious diseases. *The Lancet infectious diseases*. 2001; *1*(5): 345-353.

[3] Morens, D. M., Folkers, G. K., Fauci, A. S. The challenge of emerging and re-emerging infectious diseases. *Nature*. *2004, 430*(6996): 242-249.

[4] Adhikari, S. P., Meng, S., Wu, Y. J., Mao, Y. P., Ye, R. X., Wang, Q. Z., Zhou, H. Epidemiology, causes, clinical manifestation and diagnosis, prevention and control of coronavirus disease (COVID-19) during the early outbreak period: a scoping review. *Infectious diseases of poverty*.2020; *9*(1): 1-12.

[5] Zhou, J. B., Ma, C. X., Dong, S., ZHANG, M. J. Unconventional prevention strategies for urban public transport in the COVID-19 epidemic: taking Ningbo City as a case study. *China Journal of Highway and Transport*. 2020; *33*(12): 1-10.

[6] Ge, Z. Y., Yang, L. M., Xia, J. J., Fu, X. H., Zhang, Y. Z. Possible aerosol transmission of COVID-19 and special precautions in dentistry. *Journal of Zhejiang University-SCIENCE B*, 2020; 2020:1-8.

[7] Zeng, W., Chen, P., Nakamura, H., Iryo-Asano, M. Application of social force model to pedestrian behavior analysis at signalized crosswalk. *Transportation Research Part C: Emerging Technologies*, 2014; *40*: 143-159.

[8] Guo, Y., Sayed, T., Zheng, L., Essa, M. An extreme value theory based approach for calibration of microsimulation models for safety analysis. *Simulation Modelling Practice and Theory*. 2021; *106*: 102172.

[9] Zeng, W., Chen, P., Yu, G., Wang, Y. Specification and calibration of a microscopic model for pedestrian dynamic simulation at signalized intersections: A hybrid approach. *Transportation Research Part C: Emerging Technologies*. 2017; *80*: 37-70.

[10] Guo, Y., Liu, P., Wu, Y., Chen, J. Evaluating how right-turn treatments affect right-turn-on-red conflicts at signalized intersections. *Journal of Transportation Safety & Security*. 2020; *12*(3): 419-440.

2. Please polish the quality of English language.

- **Thank you for your comments. A thorough proofreading and language revision was made to improve the readability of the manuscript.**
- **The paper " Epidemic Spread Simulation in an Area with a High-density Crowd Using a SEIR-Based Model " by Dr. Jibiao Zhou, Sheng Dong, Changxi Ma, et al., has been edited by Native speakers for English language, grammar, punctuation, and spelling by AJE Language Editing Services from February 24th to March 5th, 2021, the certificate of editing is as follows,**

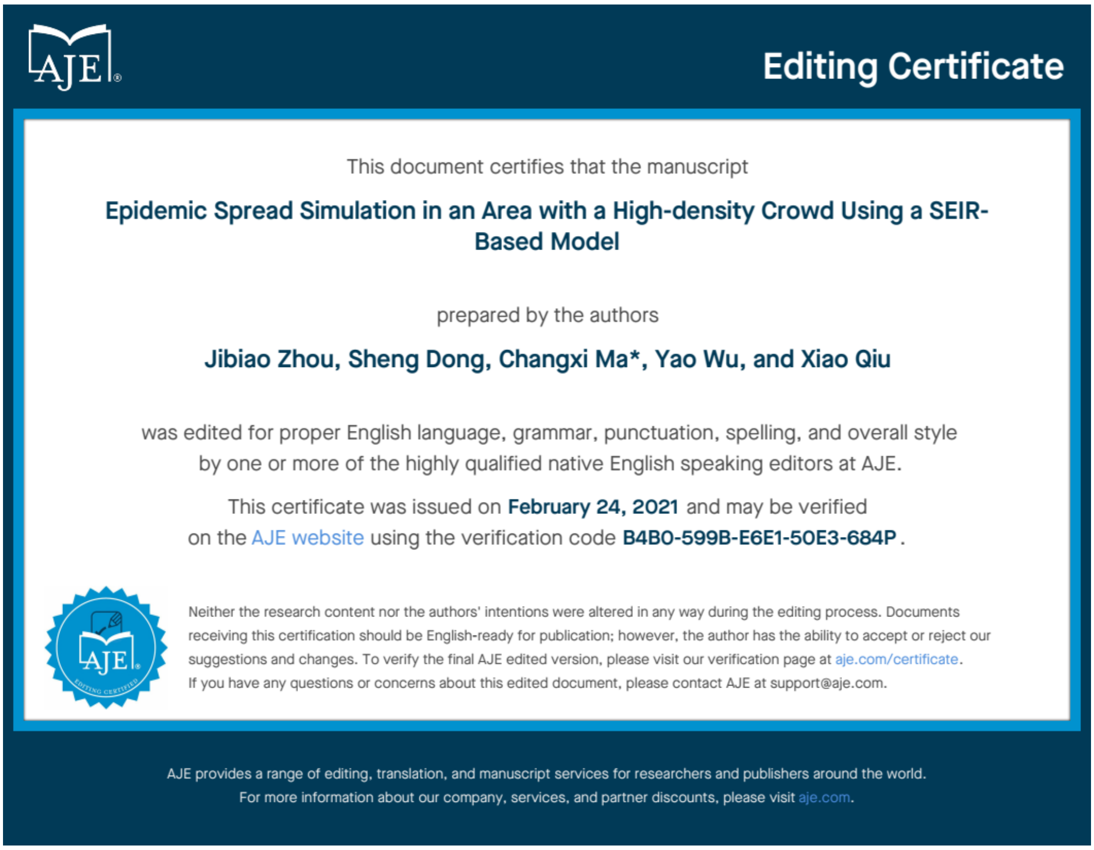

AJE

Editing Certificate

This document certifies that the manuscript

Epidemic Spread Simulation in an Area with a High-density Crowd Using a SEIR-Based Model

prepared by the authors

Jibiao Zhou, Sheng Dong, Changxi Ma*, Yao Wu, and Xiao Qiu

was edited for proper English language, grammar, punctuation, spelling, and overall style by one or more of the highly qualified native English speaking editors at AJE.

This certificate was issued on **February 24, 2021** and may be verified on the AJE website using the verification code **B4B0-599B-E6E1-50E3-684P**.

Neither the research content nor the authors' intentions were altered in any way during the editing process. Documents receiving this certification should be English-ready for publication; however, the author has the ability to accept or reject our suggestions and changes. To verify the final AJE edited version, please visit our verification page at aje.com/certificate. If you have any questions or concerns about this edited document, please contact AJE at support@aje.com.

AJE provides a range of editing, translation, and manuscript services for researchers and publishers around the world. For more information about our company, services, and partner discounts, please visit aje.com.

Reviewer #3

The article contains information technical and innovative that justifies its publication. The problem addressed is current and has technical relevance, which makes it significant. The paper is well organized and convincing. The simulation-based methodology is described as comprehensively. Interpretations and conclusions are justified by the results. The paper is well organized and convincing. My recommendations are:

1. The abstract can be rewritten to be more meaningful. The authors should add more details about their results in the abstract.

- **Many thanks for your comment. The Abstract and Literature review chapter are modified as your suggestions, see Line 5-21, Page 2; Line 4-22, Page 3-6.**

- **This is a good point. Understanding the spread of infectious diseases is an extremely essential step to preventing them. Thus, correct modeling and simulation approaches are critical for elucidating the transmission of infectious diseases and improving the control of epidemics. The primary objective of this study is to simulate the spread of communicable diseases in an urban rail transit station. Data were collected by a field investigation in the city of Ningbo, China. A SEIR-based model was developed to simulate the spread of infectious diseases in Tianyi station, considering four groups of passengers (susceptible, exposed, infected, and recovered) and a 14-day incubation period. Based on the historical data of infectious diseases, the parameters of the SEIR infectious disease model were clarified, and a sensitivity analysis of the parameters was also performed. The results showed that the contact rate (CR), infectivity (I), and average illness duration (AID) were positively correlated with the number of infections. It was also found that the length of the average incubation time (AIT) was positively correlated with the number of exposed individuals and negatively correlated with the number of infectors. These simulation results provide support for the validity and reliability of using the SEIR model in studies of the spread of epidemics and facilitate the development of effective measures to prevent and control an epidemic.**

2. Running a SEIR model for a 1,000 population without clear definition of the specifics of the problem is useless.

- **Many thanks for your comment. We have added a detailed explanation for 1,000 population, see Line 6-12, Page 14.**
- **There are 4 entrances and exits at Tingyi Station, and 4 entrances and exits are open under normal circumstances. During the survey time, we took 15 minutes as a time interval to count the flow of pedestrians entering the station during working days and non-working days respectively, and**

finally summarized them in units of 1 hour, as shown in Table 1. Hence, for the convenience of simulation statistics, the number of simulated people per unit hour was set to 1000.

Table 1 Survey results in Tianyi station

Weekend		Working-day	
Time	Number	Time	Number
07:00—08:00	1031	07:00—08:00	1221
08:00—09:00	1131	08:00—09:00	1341
09:00—10:00	899	09:00—10:00	789
10:00—11:00	932	10:00—11:00	821
11:00—12:00	899	11:00—12:00	841
12:00—13:00	1021	12:00—13:00	1021
13:00—14:00	1031	13:00—14:00	942
14:00—15:00	947	14:00—15:00	854
15:00—16:00	943	15:00—16:00	765
16:00—17:00	1034	16:00—17:00	1147
17:00—18:00	1241	17:00—18:00	1353
18:00—19:00	931	18:00—19:00	865
19:00—20:00	945	19:00—20:00	798
20:00—21:00	849	20:00—21:00	776

3. The calibration of the model parameter is not clearly described. Please check.

- **Double thanks for your comment. We have added more detailed description for our data set, as shown in Table 3.**

Table 3. Parameter values after experimental calibration

Parameter	Description	Value	Unit	Data source
N	total population	1,000	person	Average hourly cross-sectional headcount for field research
t	time	10-20	*d*	1s is used in the simulation to represent 1d
v	perturbation factor	79.34%	—	Effectiveness of protection after individual vaccination
μ	natural mortality rate	$2*10^{-5}$	person/unit time	Global natural mortality, as of March 2021
β	infection rate	$6.93*10^{-9}$	person/unit time	MCMC parameter estimation[35]
γ	recovery rate	0.12	person/unit time	MCMC parameter estimation[35]
σ	the rate of moving from exposure to infection	1/5.7	person/unit time	In the actual epidemic, the inverse of the incubation period

Note: MCMC for Markov Chain Monte Carlo.
